# Proteomics-Guided Discovery of Candidate Enzymes for Degradation of a Rare Pyruvylated Glycosaminogalactan by a Newly Isolated *Luteolibacter* Strain

**DOI:** 10.1021/acs.jproteome.6c00586

**Published:** 2026-09-29

**Authors:** Victor Daisuke Kietzmann, Pascal M. Mrozek, G. Jacob Boehlich, Ronja M. Sandholm, Sabina L. La Rosa, Bjørge Westereng, Gustav Vaaje-Kolstad

**Affiliations:** Faculty of Chemistry, Biotechnology and Food Science, 56625 Norwegian University of Life Sciences, 1432 Ås, Norway

**Keywords:** Proteomics, Mass Spectrometry, Exopolysaccharides, Enzyme Discovery, Size-Exclusion Chromatography, Carbohydrate-Active Enzymes, Differential Expression

## Abstract

Bacterial exopolysaccharides (EPS) display extensive structural diversity arising from variation in monosaccharide composition, glycosidic linkages, and non-carbohydrate modifications. However, the enzymes responsible for degrading many EPS structures remain unknown, reflecting the limited coverage of current carbohydrate-active enzyme (CAZyme) families. Here, we investigated the microbial degradation of EPS_*Lp*_, a high-molecular-mass glycosaminogalactan from *Lactiplantibacillus pentosus* KW1 containing the uncommon GalNAcβ1 → 2­(4,6-*O*-*R*-pyruvyl)­Gal motif. Enrichment cultivation with EPS_*Lp*_ as the main carbon source led to the isolation of *Luteolibacter* sp. VK1, a novel strain capable of utilizing this biopolymer. Size-exclusion chromatography and MALDI-TOF mass spectrometry showed that VK1 depolymerizes EPS_*Lp*_ into defined oligosaccharides, consistent with endo-acting cleavage followed by further processing. By combining genomics, AlphaFold-based structural annotation and comparative proteomics, we identified candidate CAZymes potentially involved in EPS_*Lp*_ catabolism. Notably, a secreted GH123 protein and a distantly related GH123-like protein were strongly associated with EPS_*Lp*_-dependent growth, suggesting that this enzyme family, or related structural homologues, may contribute to extracellular EPS_*Lp*_ depolymerization. Together, these findings support a candidate pathway for degradation of a structurally complex, pyruvylated bacterial glycan and highlight potentially novel EPS-active enzymes whose proposed roles, predicted through bioinformatic analyses, await experimental validation.

## Graphical abstract

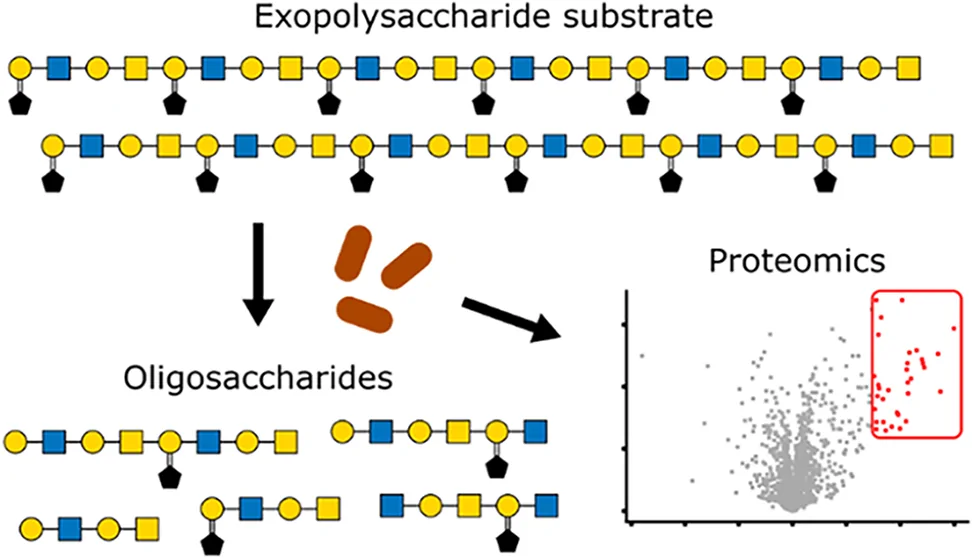

## Introduction

Bacterial exopolysaccharides (EPS) are a diverse class of high-molecular-mass glycans that play central roles in microbial ecology, including protective functions, biofilm formation, and interspecies interactions.^1–5^ They display a remarkable structural diversity, which arises from the large combinatorial space of monosaccharides, glycosidic linkages, and chemical modifications such as acetylation, sulfation, and pyruvylation.^6–9^ As a result, EPS often exhibit highly complex architectures that can substantially differ even between closely related organisms. One such example is the lactic acid bacterium species *Lactiplantibacillus pentosus*, for which four strains show completely different structures.^10–12^ While this large structural variability enables the functional versatility of EPS, it also presents a considerable challenge for their enzymatic degradation.^13^

In natural environments, EPS are subject to degradation by microbial communities through the action of carbohydrate-active enzymes (CAZymes), particularly glycoside hydrolases (GHs).^14^ These enzymes deconstruct complex polysaccharides into metabolizable oligo- and monosaccharides in a coordinated manner. Endo-acting GHs rapidly reduce polymer size by cleaving internal glycosidic linkages, while exo-acting enzymes progressively remove terminal units. Polysaccharides with high structural complexity typically require synergistic action of multiple, complementary enzymes for efficient degradation. However, uncommon linkages and chemical modifications, especially in the polysaccharide backbone, can often render polysaccharides recalcitrant to degradation.

Despite the rapid expansion of the CAZy database, which categorizes and organizes all CAZymes into distinct classes and families, the ability to predict enzymatic activities towards complex EPS remains limited.^15,16^ This is particularly true for polysaccharides containing rare glycosidic linkages or noncanonical modifications, suggesting the existence of enzyme families or catalytic mechanisms yet to be characterized. Identifying these activities is essential for advancing our understanding of microbial carbon and nitrogen cycling and may contribute to the development of biocatalysts with potential applications in biotechnology.^17,18^

One such complex polysaccharide is the high-molecular-mass glycosaminogalactan (>800 kDa) from the lactic acid bacterium *L. pentosus* KW1 (Figure 1; hereafter referred to as EPS_*Lp*_).^10^ This heteropolysaccharide contains several unusual glycosidic linkages with limited predicted CAZyme coverage, including the rare GalNAcβ1 → 2­(4,6-O-*R*-pyruvyl)­Gal motif.^14^ The structural complexity is increased further by the presence of pyruvylated residues, which may affect both enzyme recognition and catalytic efficiency. Notably, current CAZyme annotations provide no clear endo-acting enzyme for any of the linkages in EPS_*Lp*_. Therefore, EPS_*Lp*_ represents a well-suited substrate to probe unexplored enzymatic activities and expand CAZyme classifications.

**Figure 1:**
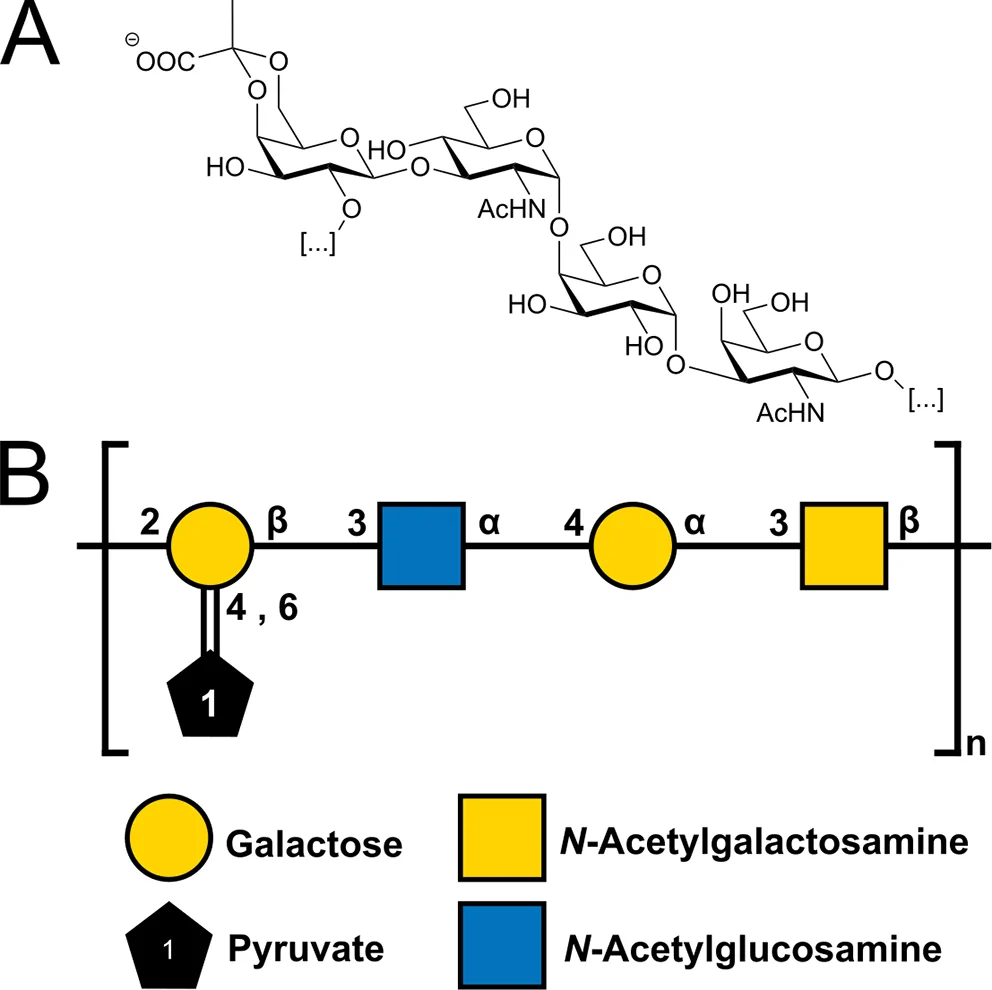
Structure of EPS_*Lp*_, the high-molecular-mass exopolysaccharide from *L. pentosus* KW1.^10^ (A) Skeletal formula and (B) Structure nomenclature for glycans.

In the present study, we employed enrichment cultures using EPS_*Lp*_ as main carbon source to isolate a novel bacterium capable of metabolizing EPS_*Lp*_. The resulting strain, *Luteolibacter* sp. VK1, was subjected to whole-genome sequencing and comparative proteomic analysis to identify enzymes associated with EPS_*Lp*_ catabolism. By integrating these omics data with biochemical degradation assays, we identified candidate EPS_*Lp*_-active enzymes and propose a working model for EPS_*Lp*_ depolymerization and downstream processing. These findings provide new insights into microbial utilization of complex polysaccharides and highlight the potential for discovering uncharacterized CAZyme activities.

## Methods

### Preparation of EPS_*Lp*_-Containing Minimal Medium

A modified M9 minimal salt medium supplemented with a mixture of EPS_*Lp*_ and two co-purified wall teichoic acids in similar amounts (named WTA-A and WTA-B) as main carbon source was used for all growth experiments (hereafter named “M9 EPS_*Lp*_-WTA”). Each liter of M9 EPS_*Lp*_-WTA contained 6 g/L EPS_*Lp*_-WTA mix, 6 g/L Na_2_HPO_4_, 3 g/L KH_2_PO_4_, 0.5 g/L NaCl, 0.5 g/L NH_4_Cl, 0.12 g/L MgSO_4_, 33 mg/L CaCl_2_, 1 mg/L Biotin, 1 mg/L Thiamin and 10 mL of trace element solution (TES). The TES contained 5 g/L EDTA, 0.83 g/L FeCl_3_ · 6H_2_O, 84 mg/L ZnCl_2_, 13 mg/L CuCl_2_·2H_2_O, 10 mg/L CoCl_2_·6H_2_O, 10 mg/L H_3_BO_3_, 1.6 mg/L MnCl_2_·6H_2_O. The mixture of EPS_*Lp*_ and WTAs (referred to as “EPS_*Lp*_-WTA mix”) was obtained as previously described.^10^

### Enrichment of Microorganisms Capable of EPS_*Lp*_ Metabolism

Microorganisms able to degrade and metabolize EPS_*Lp*_ were obtained by inoculating M9 EPS_*Lp*_-WTA with a panel of environmental and food-derived inocula (Table S1). In detail, the inocula were prepared by suspending 100 mg solid sample in 1 mL sterile M9 minimal salt medium (M9). The samples were briefly sonicated using a Bransonic 3510E-DTH Ultrasonic Cleaner (Branson Ultrasonics Corporation) for 1 min and centrifuged and 1 μL of the resulting liquids was used to inoculate aliquots of 100 μL of M9 EPS_*Lp*_-WTA. The inoculated media were incubated at 25 °C with shaking (200 rpm) in a 96-well microtiter plate and growth was monitored by both an increase in OD_600_ and visual inspection. Enrichments were incubated for around 2 weeks and subjected to two rounds of re-propagation before strain isolation.

### Isolation and Identification of Microorganisms Capable of Metabolizing EPS_*Lp*_

1 μL of culture enriched for EPS_*Lp*_-degrading communities was streaked out onto an LB agar plate and incubated for 2 days at room temperature. A minimum of one of each morphotype was picked and transferred back into 100 μL of M9 EPS_*Lp*_-WTA, incubated for up to 3 days at 25 °C with shaking (200 rpm) and analyzed by size-exclusion chromatography (SEC). Cultures showing EPS_*Lp*_ or WTA degradation were subjected to 16S rRNA sequencing (as detailed below) to assess culture purity. This procedure was repeated until EPS_*Lp*_- or WTA-degrading isolates were obtained.

### 16S Ribosomal RNA Gene Sequencing

PCR was performed using Q5 Hot Start High-Fidelity 2X Master Mix (New England Biolabs, USA) and 16S rRNA gene specific primers 27F (5′-AGAGTTTGATCMTGGCTCAG-3′) and 1492R (5′-CGGTTACCTTGTTACGACTT-3′).^19^ PCR conditions were: 95 °C for 5 min; 35 cycles of 95 °C for 20 s, 55 °C for 30 s, 72 °C for 50 s; followed by 72 °C for 5 min. PCR cleanup was performed using a NucleoSpin Gel and PCR Clean-up kit (Macherey-Nagel, Germany) and standard procedures. Purified PCR products were submitted with the 27F and 1492R amplification primers to Eurofins Genomics (Germany) for Sanger sequencing.

### Genome Sequencing, Assembly and Annotation

Isolates able to metabolize EPS_*Lp*_, according to polysaccharide depletion analysis by size exclusion chromatography (SEC) (see below for method details), were cultured in Luria Bertani broth for 48 h at 22 °C with shaking (200 rpm). Genomic DNA was extracted using the DNeasy PowerSoil Pro kit (QIAGEN, Germany) following the manufacturer’s instructions. DNA concentration and purity were measured using a NanoDrop One spectrophotometer and a Qubit 3.0 fluorometer with the dsDNA High Sensitivity assay kit (Thermo Fisher Scientific, USA). DNA quality was assessed by a 0.35% agarose gel electrophoresis on a Bio-Rad Gel Doc EZ Imager. High molecular weight DNA fragments were collected using a BluePippin system (Sage Science, USA).

Sequencing libraries were prepared using the Native Barcoding kit SQK-NBD114.24 (Oxford Nanopore Technologies, UK), followed by Oxford Nanopore 1D Genomic DNA by ligation sequencing kit SQK-LSK109 (Oxford Nanopore Technologies, UK), according to the manufacturer’s instructions. Samples were sequenced on an R10.4 flow cell using a PromethION device (Oxford Nanopore Technologies, UK). POD5 files were base-called and demultiplexed with Dorado v0.5.0 (https://github.com/nanoporetech/dorado), using the super-accurate model (config: dna_r10.4.1_e8.2_400bps_sup@v4.3.0). Library preparation and sequencing were carried out by Biomarker Technologies GmbH (Münster, Germany).

Raw reads were quality-filtered with FiltLong v0.2.1 (https://github.com/rrwick/Filtlong) using the parameters --min_length 1000, --keep_percent 90 and assembled using Flye v2.9.2 (--nano-raw, default settings).^20^ Contigs were initially polished with Racon (−include-unpolished) (https://github.com/isovic/racon), followed by 2 rounds of Medaka (−model r941_min_fast_g303_model.hdf5) (https://github.com/nanoporetech/medaka). Assembly completeness was evaluated with BUSCO v.6.0.0, using the verrucomicrobiota_odb12 database.^21^ GTDB-Tk with the GTDB database release 226 was used to determine the taxonomic assignment.^22^ Functional annotations were obtained using DRAM v1.5.0 with the Uniref90, PFAM-A, KOfam and dbCAN-V11 databases (all downloaded in April 2025).^23^

### Substrate-Induced Secretome/Proteome Activity Assay

The potential activity of secreted or cytoplasmic/periplasmic proteins from the EPS_*Lp*_-consuming bacterium *Luteolibacter* sp. VK1 identified in this study, was probed by obtaining crude protein extracts, incubating these with EPS_*Lp*_-WTA mix and analyzing putative reaction products. In detail, three glass tubes containing 5 mL M9 EPS_*Lp*_-WTA were inoculated with 1 μL of *Luteolibacter* sp. VK1 cryostock and grown in a shaking incubator at 25 °C with shaking (200 rpm). OD measurements and 100 μL samples were taken at regular intervals. After 32 h of incubation the cultures were pooled to obtain sufficient sample material for downstream analysis and centrifuged at 4500*g* and 4 °C for 10 min. The resulting supernatant containing putatively secreted enzymes (secreted protein fraction) was kept on ice for later use. The remaining cell pellet was washed three times by careful resuspension in 15 mL of lysis buffer (20 mM Tris-HCl pH 7, 100 mM NaCl) and centrifuging as described above while discarding the supernatant between washing steps. The washed cell pellet was finally resuspended in 15 mL lysis buffer and sonicated in 5 second intervals for 10 min with an Ultrasonic processor (Qsonica, USA) to lyse the cells. The lysate was clarified by centrifuging and decanting as described above, this time collecting the supernatant (intracellular protein fraction, i.e., containing putative enzymes from both the periplasm and cytoplasm). Both samples were concentrated and buffer-exchanged in lysis buffer three times using Amicon 3 kDa MWCO centrifugal filters (Merck, Germany) by reducing retentate volume to 1 mL and filling up to 15 mL with lysis buffer. Finally, both samples were reduced to 1 mL a final time and sterile filtered using Millex GV 0.22 μm syringe filters (Merck, Germany). Both samples were used in assays immediately after preparation by adding 67 μL of lysate or supernatant sample to 933 μL reaction buffer (20 mM Tris-HCl pH 7, 100 mM NaCl) containing 1.5 mg/mL EPS_*Lp*_ and WTA-A and -B. After mixing the reaction mixture was incubated in a Thermomixer C (Eppendorf, Germany) at 25 °C and 500 rpm. Samples were taken at regular intervals and analyzed using SEC and MALDI-ToF MS (see below for detailed protocols).

### Size-Exclusion Chromatography (SEC)

Samples obtained from the enrichment cultures, isolation steps and substrate-induced secretome/proteome assay were prepared for SEC analysis by mixing 20 μL of sample with 80 μL of deionized water, heating at 95 °C for 5 min, centrifuging at 11,000*g* for 5 min and filtering through 0.22 μm filters. Analysis was performed at room temperature using a Yarra SEC-4000 column and a Yarra SEC-2000 column (300 × 7.8 mm, 3 μm; Phenomenex, USA), coupled in series to a Dionex UltiMate 3000 HPLC system (Thermo Fisher Scientific, USA). The mobile phase consisted of 0.1 M Na_2_SO_4_, and the flow rate was set to 0.5 mL/min. Injection volumes were 20 μL. Detection was performed by UV 210 nm using a Dionex UltiMate 3000 VWD-3000 detector (Thermo Fisher Scientific, USA) and refractive index (RI) using a RefractoMax 520 RI-Detector (ERC Inc., Japan).

### Matrix-Assisted Laser Desorption/Ionization—Time-of-Flight Mass Spectrometry (MALDI-ToF MS)

Samples obtained from the enrichment cultures and substrate-induced secretome/proteome assay were prepared for MADLI-ToF MS analysis by mixing 2 μL of sample with 18 μL of deionized water, heating at 95 °C for 5 min and centrifuging at 11,000*g* for 5 min. 1 μL of sample was spotted on a target plate with 2 μL of 9 mg/mL 2,5-dihydroxybenzoic acid/ACN (70/30, v/v) and dried under a stream of warm air. Samples were analyzed using a Bruker UltrafleXtreme instrument (Billerica, USA). The mass range used for analysis was between 300 and 4000 Da.

### Genome Analysis

All CAZymes annotated in the genome were examined with respect to their annotated CAZy family classifications and associated activities using the CAZy database.^15,16^ The database was also queried using CAZac activity descriptors corresponding to all linkages present in the EPS_*Lp*_ backbone, including broad annotations based solely on assays using chromogenic model substrates like *p*NP-glycosides.^14^ CBMs were also examined with respect to their reported binding specificities whenever present within the same protein. Signal peptides were identified by analyzing the amino acid sequences using SignalP 6.0 (web server, default settings).^24^

### Proteomics

Pre-cultures of *Luteolibacter* sp. VK1 were grown for 3 days at 25 °C with shaking (200 rpm) in 5 mL modified tryptic soy broth (TSB, Merck, Germany) supplemented with either EPS_*Lp*_-WTA mix or glucose. Each liter of diluted TSB contained 3.25 g pancreatic digest of casein, 0.75 g papain digest of soybean meal, 1.25 g NaCl, 0.7 g K_2_HPO_4_ and 6 g of either EPS_*Lp*_-WTA mix (TSB-EPS) or glucose (TSB-Glc). After three pass-throughs, 3 replicates of TSB-EPS and 9 replicates of TSB-Glc were inoculated with 50 μL and 100 μL of pre-culture and incubated for 15.5 and 17 h, respectively, while monitoring optical density. Due to the lower final OD in TSB-Glc, groups of three replicates of the TSB-Glc cultures were pooled to obtain three 15 mL cultures for harvesting and further processing. After incubation and pooling, cultures were centrifuged at 4696*g* and 4 °C for 15 min, decanted, and the cells stored at −80 °C until further use.

For proteomics analysis, cell pellets were resuspended in 300 μL of 4% SDS (w/v) and lysed by bead beating 3 times at 4.5 m/s for 1 min using a FastPrep-24 homogenizer (MP Biomedicals, USA), with 5 min cooling between each cycle. Following lysis, the samples were centrifuged at 16,000*g* for 10 min and 250 μL of the supernatants were collected and used for protein extraction using the Wessel-Fluegge precipitation method.^25^ The precipitated protein was air-dried in a laminar flow hood. Dried protein pellets were resuspended in 20 μL denaturing sample buffer (0.25 M DTT, 50% glycerol v/v, 0.225 M Tris-HCl pH 6.8, 0.05% bromophenol blue v/v, 5% SDS w/v), incubated at 95 °C for 5 min and run on SDS-PAGE using Any kD Mini-PROTEAN TGX Stain-Free polyacrylamide gels (Bio-Rad Laboratories, USA) at 270 V for 3 min. SDS-PAGE gels were stained using Coomassie staining solution (0.1% Coomassie blue R250 (w/v), 40% ethanol (v/v), 10% acetic acid (v/v)) and the protein bands were excised for reduction and alkylation. Reduction was performed by adding 50 μL of DTT solution (10 mM Dithiothreitol, 100 mM NH_4_CO_3_), incubating for 30 min at 56 °C and removing the DTT solution. Proteins were alkylated by adding 50 μL of IAA solution (55 mM Iodoacetamide, 100 mM NH_4_CO_3_), incubating for 30 min at RT in the dark and removing the IAA solution. After dehydration of the gel cubes with acetonitrile and air-drying, in-gel digestion was performed by adding 30 μL of 10 ng/μL trypsin per sample, followed by incubation at 37 °C for 20 h. Peptides were extracted from gel cubes by sonication for 10 min at full power in a water bath in 0.1% TFA. The peptides in the solution were purified using C18 ZipTips (Millipore, USA), cleaned with 10 μL of 0.1% TFA and eluted using 10 μL of an elution solution containing 70% acetonitrile and 0.1% TFA. Lastly, the peptides were dried using a Concentrator Plus (Eppendorf, Germany).

The peptides were analyzed by nanoLC-MS/MS as described previously, using a Q-Exactive hybrid quadrupole orbitrap mass spectrometer (Thermo Fisher Scientific, USA), with several adjustments.^26^ Dried peptides were dissolved in 1% (v/v) formic acid, 0.05% (v/v) acetonitrile in water and injected into an Ultimate 3000 nano ultra high-performance liquid chromatography system (Dionex, USA) connected to a Q-Exactive quadrupole-orbitrap mass spectrometer (Thermo Fisher Scientific, USA) equipped with a nano-electrospray ion source. Chromatographic separation was done using a nanoViper Acclaim PepMap 100 column (C18, 3 μm, 100 Å, 500 mm × 0.075 mm) (Dionex, USA). The flow rate was 300 nL/min, and the solvent gradient was 12% B to 43% B in 93 min, followed by further increase to 90% B in 6 min. Solvent A was 0.1% (v/v) formic acid in water and solvent B was 80% (v/v) acetonitrile, 19.92% water (v/v), 0.08% (v/v) formic acid. The mass spectrometer was operated in data-dependent mode to switch automatically between orbitrap-MS and higher-energy collisional dissociation (HCD) orbitrap-MS/MS acquisition. The resolution was *R* = 70,000 and *R* = 35,000 for MS and MS/MS, respectively. For optimal acquisition of MS/MS spectra, automatic gain control (AGC) target values were set to 1,000,000 charges or a maximum injection time of 128 ms was allowed. Data dependent analysis was applied to isolate and fragment the 10 most intense ions at any given time throughout the chromatographic elution. These ion precursors were then excluded for further fragmentation for 20 seconds.

MS data analysis was performed as described previously, with small adjustments.^27^ In brief, raw MS data files were processed using the FragPipe suite v17.1, incorporating MSFragger v3.4 and Philosopher v4.2.1.^28,29^ The protein database was built using the *Luteolibacter* sp. VK1 genome, including reversed decoy sequences. Trypsin was specified as digestion enzyme, and one missed cleavage was allowed. Carbamidomethylation of cysteine residues was set as a fixed modification, and methionine oxidation and protein *N*-terminal acetylation were allowed as variable modifications. Proteins were filtered to 1% FDR, and a protein was considered present when detected in at least two of the three replicates from either substrate used. The data analysis was performed using Perseus v1.6.13.0.^30^ Protein abundances were represented as relative log2-transformed label-free quantification (LFQ) values. Missing values were imputed from a normal distribution using a width of 0.3 and down shifted 1.8 standard deviations from the original distribution in total matrix mode. Differential abundance analysis was performed using an unpaired two-tailed Student’s t-test with a permutation-based FDR set to 0.05. Heat map representations were generated using Perseus v1.6.13.0. Proteins with a p-value below 0.01 and a log_2_ fold change above 3 were considered differentially abundant in TSB-EPS cultures.

### Structure-Based Protein Annotation

Proteins identified as potential candidates for EPS_*Lp*_ degradation from the proteomics experiment, i.e., either upregulated or uniquely expressed in M9 EPS_*Lp*_-WTA were subjected to structure-based functional prediction. First, structure models of the candidate proteins were obtained using the AlphaFold3 ^31^ server with default parameters for single-chain predictions using their amino acid sequences obtained from the genome sequence. Next, the AlphaFold-predicted models were submitted as queries to the FoldSeek structure alignment tool^32^ and searched against the PDB structure database to identify structurally similar proteins. The resulting structural alignments were filtered based on *E*-values (all >0.01 were discarded) and alignment coverage (alignments with <50% coverage were discarded). When available, the 10 hits with lowest *E*-values were evaluated and used to aggregate the most prevalent annotations. If no alignments of sufficient quality were available, the search was repeated against the AlphaFold databases and the resulting alignments used instead. Further refinement of proteins was based on the presence of carbohydrate-related annotations for the target proteins, while proteins with high-confidence annotations unrelated to carbohydrate metabolism were excluded.

## Results and Discussion

Microbial polysaccharides display enormous structural diversity, yet they are continually degraded and recycled in natural environments. Enrichment of organisms capable of metabolizing defined polysaccharides, followed by omics-guided candidate identification and biochemical validation, therefore provides a powerful route to discovering the enzymes responsible for their degradation. Such an approach was employed in the present study, as the current literature gives few clues to the potential enzymes capable of cleaving the glycosidic bonds and substituents of EPS_*Lp*_.

The first stage of the study aimed to identify microorganisms capable of selectively degrading and utilizing EPS_*Lp*_. Because two WTAs co-purify with EPS_*Lp*_, the enrichment and isolation strategy combined OD_600_ monitoring with concomitant SEC analysis of the biopolymer substrate to verify growth-associated and selective EPS_*Lp*_ degradation. Initially, microbial growth was observed to various degrees when inoculating M9 EPS_*Lp*_-WTA with several sources of bacterial communities (Figure 2A). After two rounds of propagation, growth gradually decreased for most communities, and only one community from a compost-derived inoculum maintained significant OD_600_ (Figure 2B). SEC analysis of the polymeric substrate confirmed that the enriched community utilized both EPS_*Lp*_ and WTAs as shown by the loss of the respective peaks (Figure 2C). To facilitate downstream identification of EPS_*Lp*_-depolymerizing enzymes, we next sought to isolate individual bacterial strains capable of EPS_*Lp*_ catabolism. The EPS_*Lp*_-WTA-degrading community was subjected to repeated streaking on LB agar, followed by reinoculation of M9 EPS_*Lp*_-WTA with single colonies. Growth was monitored by OD_600_ measurements and paired with SEC-based analysis of substrate consumption. This isolation strategy identified three isolates capable of utilizing either EPS_*Lp*_ (Figure 2D, isolate 1) or WTA-A and -B. (Figure 2D, isolates 2 and 3).

**Figure 2:**
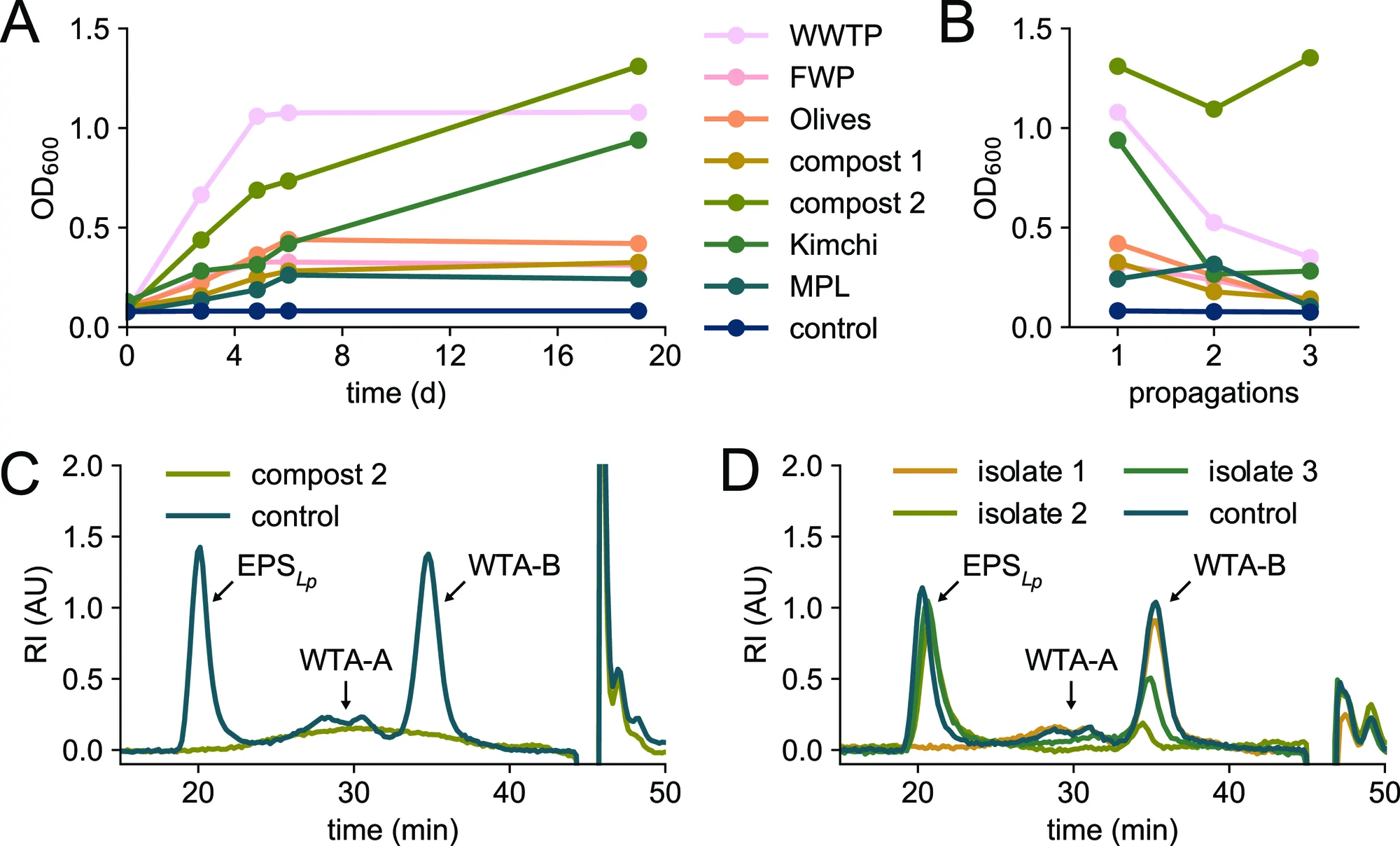
Enrichment and isolation of an EPS_*Lp*_-degrading bacterium at 25 °C with shaking (200 rpm). (A) Growth of microbial communities derived from various environmental and food-associated inocula in M9 EPS_*Lp*_-WTA. (B) Final OD_600_ values of enrichment cultures resulting from the first, second and third propagation, each measured after around 2 weeks of incubation. (C) SEC analysis of the spent culture medium resulting from growth of the enrichment culture obtained by the “compost 2” inoculum at the end of the third round of propagation. (D) SEC analysis of the spent culture medium obtained from growth of three individual isolates from the “compost 2” enrichment. WWTP = Wastewater treatment plant sample; FWP = Food waste processing plant sample; MPL = Mixed plant leaves sample.

Through genome sequencing, the two isolates utilizing WTA-A and -B were assigned to *Sphingobacterium* sp. The third isolate, which was assigned to a novel *Luteolibacter* species, designated *Luteolibacter* sp. VK1, showed stable growth and selective EPS_*Lp*_ utilization (Figure 3, panels A and B, respectively).

**Figure 3:**
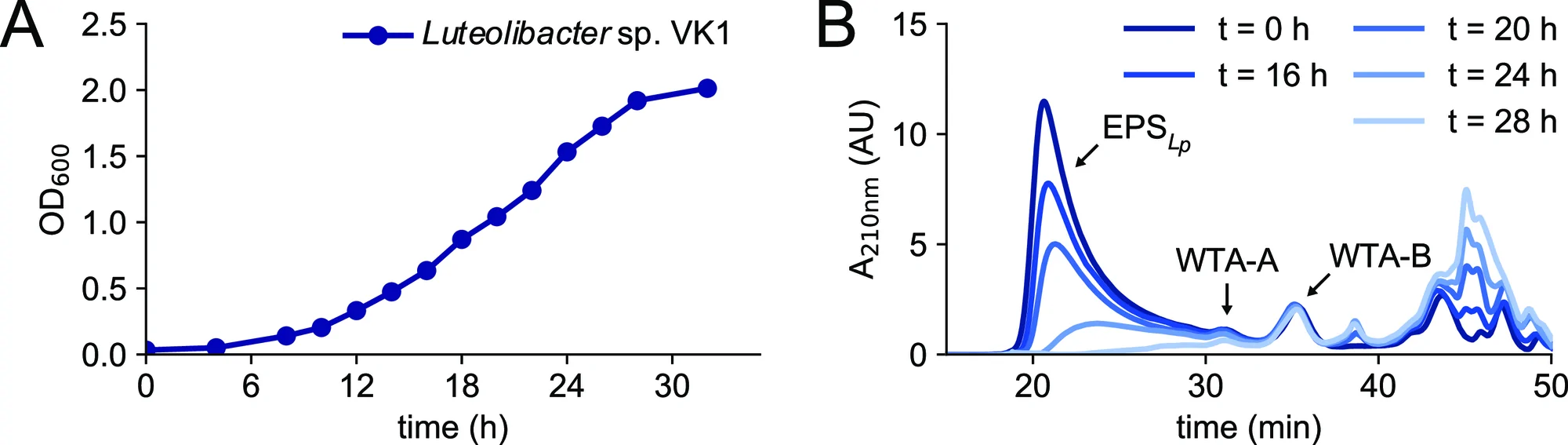
Degradation of EPS_*Lp*_ by *Luteolibacter* sp. VK1. (A) Growth curve of *Luteolibacter* sp. VK1 cultivated in M9 EPS_*Lp*_-WTA. (B) SEC profile of culture supernatant over the course of cultivation. Peaks at 21, 32, and 36 min correspond to full-length EPS_*Lp*_, WTA-A and WTA-B, respectively. Cultures were incubated at 25 °C with shaking (200 rpm) in M9 EPS_*Lp*_-WTA.

### *Luteolibacter* sp. VK1 Enzymes Hydrolyze EPS_*Lp*_

To assess the presence of secreted and intracellular enzymes capable of EPS_*Lp*_ degradation, sterile-filtered and concentrated *Luteolibacter* sp. VK1 culture supernatant and cell lysate (Figure 4A) were incubated with EPS_*Lp*_-WTA mix and monitored by SEC over time. The resulting SEC time course revealed a shift of the high-molecular-mass EPS_*Lp*_ signal towards longer retention times for both samples, even yielding resolvable individual peaks, indicating enzymatic cleavage of the polysaccharide into smaller products (Figure 4B,C).

**Figure 4:**
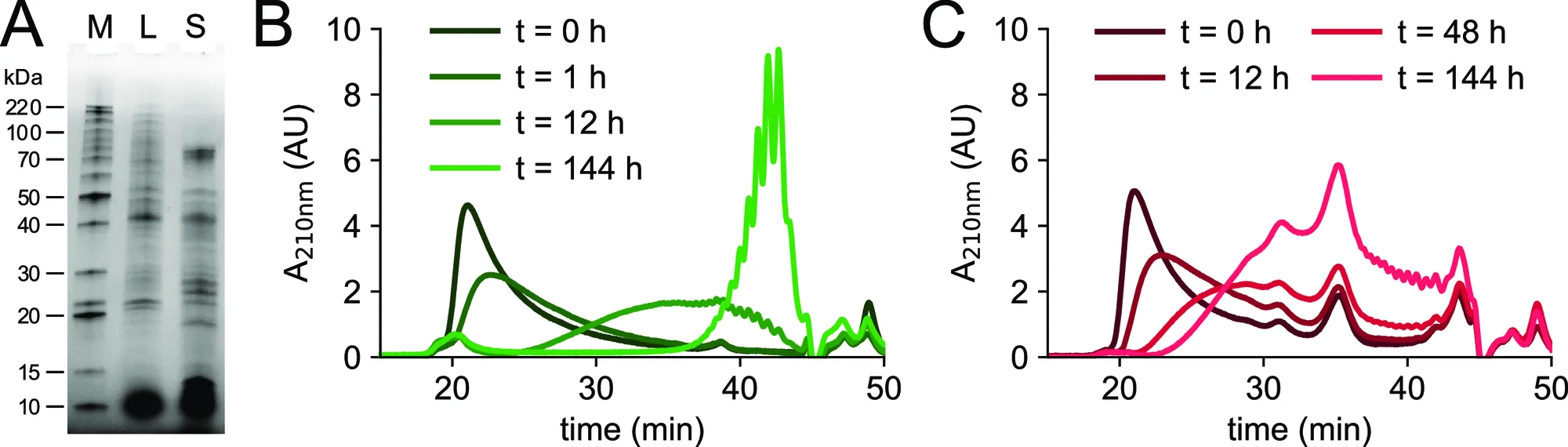
Degradation of EPS_*Lp*_ using *Luteolibacter* sp. VK1 derived protein extracts. (A) SDS-PAGE gel of protein extracts visualized using stain-free imaging. 2.5 μL of concentrated cell lysate (L) and 10 μL of concentrated culture supernatant (S) were loaded, respectively. (B) SEC profiles of EPS_*Lp*_ incubated with concentrated cell lysate. The peak at 21 min corresponds to full-length EPS_*Lp*_. (C) SEC profiles of EPS_*Lp*_ incubated with concentrated culture supernatant containing secreted proteins. Signals at 32 and 36 min originate from WTA-A and -B present in the growth medium and retained during concentration of the sample. Reactions were performed over 144 h at 25 °C with shaking (200 rpm) using 1.5 mg/mL of EPS_*Lp*_ dissolved in 20 mM Tris-HCl, 100 mM NaCl and sterile protein extract concentrations corresponding to those present in the culture at the time of harvest.

Given the relatively high apparent molecular mass of EPS_*Lp*_ (>800 kDa),^10^ the formation of a broad distribution of lower-molecular-mass species, most likely oligosaccharides, suggests the presence of one or more endo-acting enzymes targeting the EPS_*Lp*_ backbone. Exopolysaccharide degradation was observed for both lysate and culture supernatant fractions, with slower progression in the latter (Figure 4B and C). It is interesting to note that the cell lysate sample achieves almost complete degradation of EPS_*Lp*_ to oligosaccharides as this indicates the presence of an endo-acting enzyme. The present data do not allow us to determine whether this reflects true intracellular activity or activity from an enzyme released during secretion. The culture supernatant produced an EPS_*Lp*_ degradation profile resembling that of the cell lysate, indicating that EPS_*Lp*_-degrading activity was present outside the cells. This could reflect secretion of one or more endo-acting enzymes. However, SDS-PAGE analysis (Figure 4A) showed that the supernatant and lysate contained a broadly similar number of visible protein bands, whereas culture supernatants would normally be expected to contain fewer proteins than cell lysates. This suggests that substantial cell lysis may have occurred during growth or sample preparation, potentially releasing intracellular or periplasmic enzymes into the medium. The higher total protein concentration in the lysate may also explain its stronger activity. Thus, although the activity detected in the supernatant is consistent with extracellular EPS_*Lp*_ degradation, the present data do not allow secreted enzymes to be distinguished from enzymes released by cell lysis.

To obtain deeper insight into the oligosaccharide products generated during enzymatic degradation of the EPS_*Lp*_-WTA mixture, unprocessed culture supernatant and products released after incubation with cell lysate from cultures grown for 32 h (late-exponential-phase) on the same substrate were analyzed by MALDI-ToF MS. Both samples showed EPS_*Lp*_ oligosaccharides resulting from hydrolysis (Figure 5A,B), but the product profiles were different. Products generated by the culture supernatant revealed peaks corresponding to the EPS_*Lp*_ tetrasaccharide repeating unit, as well as partially modified/degraded variants thereof, including a depyruvylated species (*m/z* 771.283) that showed the most intense signal in the spectrum. Additional ions assigned to oligosaccharides containing 4, 5, 6, 7, and 8 monosaccharides were observed, each carrying a single pyruvyl group. Because cleavage by a single strictly endo-acting enzyme would be expected to generate products corresponding primarily to one or more intact tetrameric repeating units, the presence of products with non-tetrameric degrees of polymerization suggests that the culture supernatant contained additional enzymatic activities involved in processing the primary endo-cleavage products. In contrast, incubation of EPS_*Lp*_ with cell lysate produced a product profile with no substantial accumulation of depyruvylated species (Figure 5B). Instead, the dominant products corresponded to oligosaccharides comprising one or two EPS_*Lp*_ repeating units, i.e., tetrameric and octameric products carrying one and two pyruvyl groups, respectively. This shows that EPS_*Lp*_ depolymerization could be reproduced in vitro using the cell lysate, but that the product profile differed markedly from that generated by the culture supernatant. Notably, the culture supernatant yielded depyruvylated oligosaccharides together with products of non-tetrameric degrees of polymerization, suggesting additional oligosaccharide-processing activities beyond the primary endo-depolymerization step. One possible explanation is that depyruvylation represents a prerequisite step that renders EPS_*Lp*_-derived oligosaccharides accessible to further processing. In this scenario, removal of pyruvyl substituents would not merely represent a side reaction, but could be an enabling activity required for more complete EPS_*Lp*_ depolymerization.

**Figure 5:**
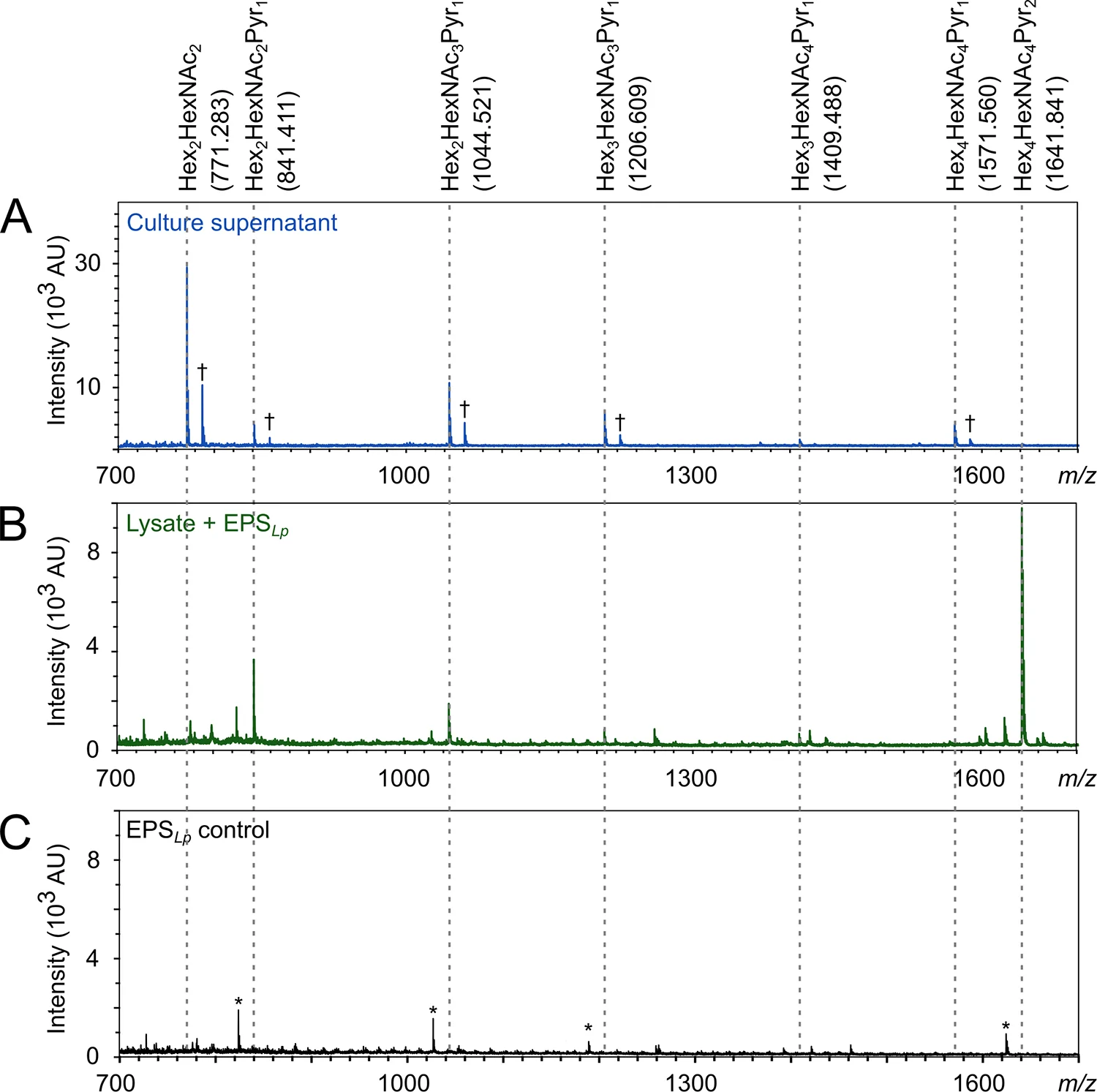
Analysis of enzymatic EPS_*Lp*_ degradation. (A) MALDI-TOF MS spectrum of unprocessed culture supernatant of *Luteolibacter* sp. VK1 grown in M9 EPS_*Lp*_-WTA sampled at 32 h incubation time. (B) MALDI-TOF spectrum of products generated by incubating purified EPS_*Lp*_ (3 g L^–1^) in 20 mM Tris-HCl, pH 7.0, 100 mM NaCl with crude protein extracts from the cell lysate of *Luteolibacter* sp. VK1 grown to late exponential phase in M9 EPS_*Lp*_-WTA. (C) EPS_*Lp*_ (3 g L^–1^) dissolved in 20 mM Tris-HCl, pH 7.0, 100 mM NaCl. Asterisks (*) highlight minor background signals corresponding to dehydrated EPS_*Lp*_-derived oligosaccharides. Daggers (†) highlight potassium adducts. Hex: Hexose; HexNAc: *N*-acetylhexosamine; Pyr: Pyruvate.

### Analysis of the *Luteolibacter* sp. VK1 CAZyme Repertoire

To gain insights into the genetic basis of the observed catabolism of EPS_*Lp*_, and to enable construction of a database for proteomic analysis of the bacterium, the genome of *Luteolibacter* sp. VK1 was analyzed. First, a genome assembly was performed using Oxford Nanopore long-read sequencing data. The assembly resulted in one circular chromosome of 4,687,387 bp, with an overall G + C content of 60.25%. The genome achieved a coverage of 100×. BUSCO-based assessment of the genome quality indicated a completeness of 99.5%. In total, 4028 genes, including 51 tRNAs and three complete rRNAs were predicted. Gene prediction and annotation were obtained using DRAM v1.5.0 with the dbCAN-V11 database and showed that *Luteolibacter* sp. VK1 possesses a large CAZyme repertoire, spanning 6 PL and 38 GH families (Table S2). A large set of GH109 and GH179 family members was identified, which include enzymes acting on *N*-acetylhexosamine-containing substrates.^33–36^ Several members of the GH1, GH2, GH3 and GH5 families were also identified, which all contain β-galactosidase activities.^37–40^

The abundance of oligosaccharides at dp4 and dp8 detected when treating EPS_*Lp*_ with VK1 cell lysate strongly suggests the presence of at least one enzyme acting within the EPS_*Lp*_ backbone. To uncover candidate enzymes responsible for these activities, we queried the CAZy database using CAZac activity descriptors^14^ to identify enzyme families reported to act on the glycosidic linkages present in EPS_*Lp*_. Notably, this analysis did not identify any CAZyme families with reported endo-acting activity toward these glycosidic linkages, suggesting that EPS_*Lp*_ degradation may involve previously uncharacterized enzymes. On the other hand, a gene encoding a member of the GH123 family (locus tag: VK1_493), which family members are known to cleave GalNAcβ1 → 3Gal and GalNAcβ1 → 4Gal bonds, was identified. Members of the GH123 family are generally annotated as exo-acting, but a recent study reports the release of oligosaccharides and an open active site cleft for some members,^41^ which suggests that some GH123 members may possess endo-activities. VK1_493 also carries an N-terminal CBM32-domain that has been reported to bind to lacto-*N*-biose (β-D-galactosyl-1,3-β-D-*N*-acetylglucosamine),^42^ a linkage found in EPS_*Lp*_. Together, these observations suggest that GH123 family enzymes may contribute to the observed depolymerization of EPS_*Lp*_.

### Proteomic Identification of Enzymes Associated with EPS_*Lp*_ Metabolism

Although analysis of the *Luteolibacter* sp. VK1 genome revealed a broad CAZyme repertoire, it provided only a theoretical basis for identifying enzymes involved in EPS_*Lp*_ degradation. Therefore, comparative proteomic analysis of cells grown on EPS_*Lp*_-WTA mix or glucose was performed to identify enzymes associated with EPS_*Lp*_ metabolism. In total, 2189 proteins were detected, of which 33 were significantly more abundant compared to glucose-grown cells, an additional 34 proteins were consistently detected exclusively in all EPS_*Lp*_-WTA mix replicates, and 27 were uniquely detected in two out of three replicates (Table S3).

To improve functional annotation beyond sequence-based methods, we generated structure predictions for all proteins uniquely regulated or more abundantly expressed when growing on the EPS_*Lp*_-WTA mix using AlphaFold3 and performed structural similarity searches using FoldSeek (Table S3). This enabled the identification of putative remote homologues, with predicted activities towards EPS_*Lp*_, that were not detectable by sequence-based annotation alone. Following manual curation based on structural similarity to proteins with suggested or established carbohydrate-related functions, we identified a final set of 22 proteins potentially involved in the EPS_*Lp*_-WTA catabolism (Figure 6A,B). Based on the culture supernatant analysis described above (Figure 3B), these are presumed to be targeting EPS_*Lp*_, although it cannot be excluded that some of these represent a response to the presence of WTA-A and -B. Most of the genes encoding these proteins are scattered throughout the genome, suggesting a more dispersed genomic organization of the degradative machinery rather than clustering within a dedicated polysaccharide utilization locus (PUL), as observed for the catabolism of other exopolysaccharides such as xanthan gum.^43–46^

**Figure 6:**
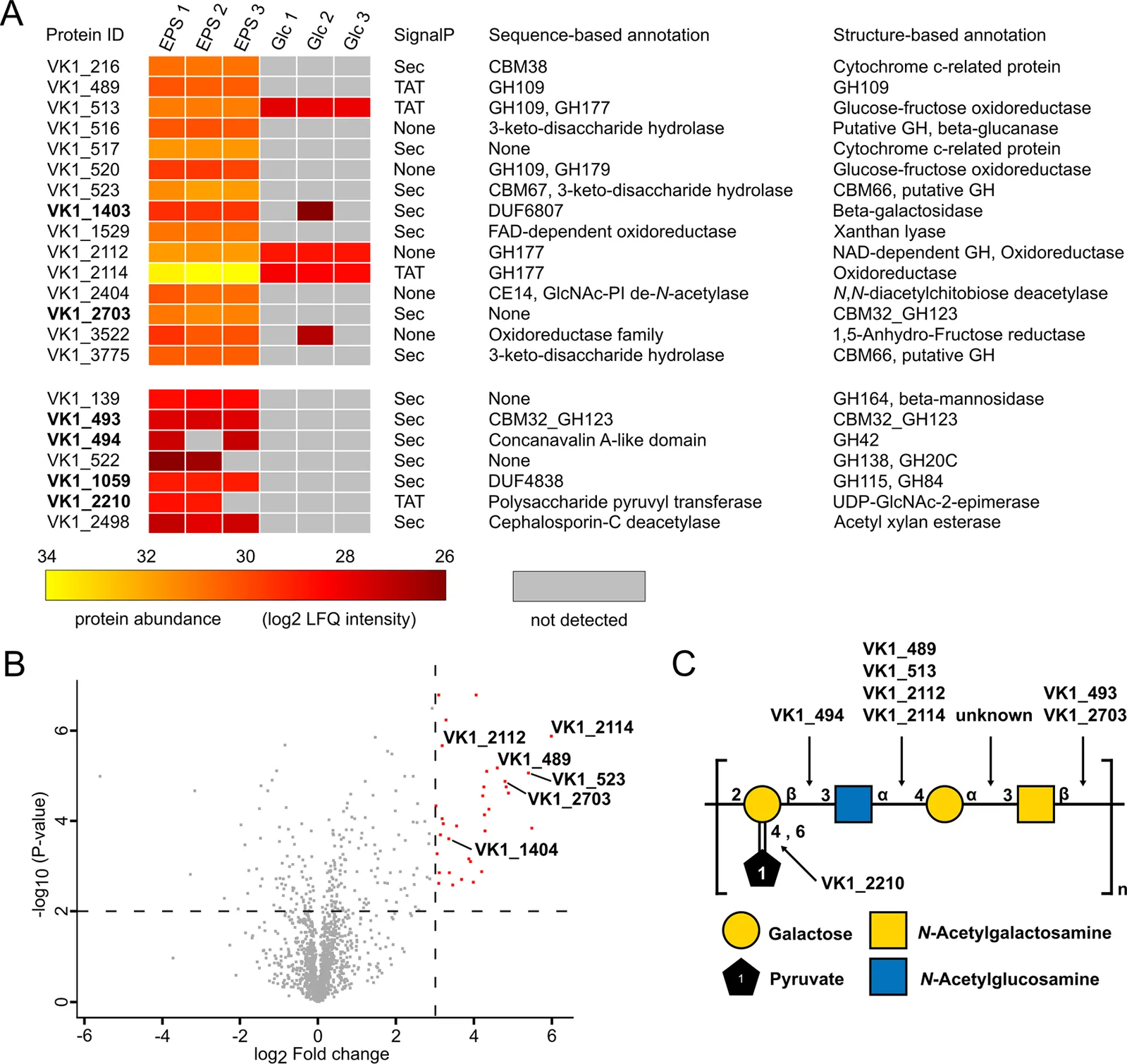
Proteomics analysis of *Luteolibacter* sp. VK1 grown in diluted tryptic soy broth supplemented with either *L. pentosus* EPS_*Lp*_-WTA mix or glucose. (A) Curated set of proteins potentially involved in EPS_*Lp*_ catabolism. Sequence- and structure-based annotations were obtained using DRAM and AF3 paired with FoldSeek, respectively. Top panel: selection of proteins that were upregulated when grown on EPS_*Lp*_-WTA. Bottom panel: curated selection of proteins that were not upregulated but uniquely detected in EPS_*Lp*_-WTA samples. (B) Volcano plot of all detected VK1 proteins, 33 of which were found to be significantly more abundant and are highlighted in red. (C) Proposed assignment of VK1 proteins directly involved in EPS_*Lp*_ catabolism.

Of the 22 proteins relevant for EPS_*Lp*_ degradation, only a few can be rationalized for cleavage of the EPS_*Lp*_ glycosidic bonds or the pyruvylation motif. For hydrolysis of the GalNAcβ1 → 2­(4,6-O-*R*-pyruvyl)­Gal bond, the genomic data already suggested the family GH123 protein, VK1_493, as a potential candidate for exo- or endo-cleavage. This protein was indeed observed as uniquely expressed when EPS_*Lp*_-WTA mix was used as a main carbon source, strengthening its putative role in the EPS_*Lp*_ catabolic pathway. Interestingly, the structure-based annotation identified a distant homolog of VK1_493, VK1_2703, which also possesses an *N*-terminal CBM32 domain and which was highly abundant in the EPS_*Lp*_-degrading proteome (Figure 6A,B). Both VK1_2703 and VK1_493 carry predicted signal peptides for Sec-mediated secretion. These findings are consistent with the involvement of the GH123 family, and related enzymes, in EPS_*Lp*_ depolymerization, likely through secretion into the extracellular environment and by cleavage of the GalNAcβ1→2­(4,6-O-*R*-pyruvyl)­Gal bond. Furthermore, of the most relevant candidates, this is the only family that is indicated to perform endo-cleavage of polysaccharides.^41^

Hydrolysis of Galβ1 → 3GlcNAc bonds has been reported for enzymes in GH families 2, 35, 42, and 112. The proteomics data showed that VK1_494, a member of the GH42 family, was uniquely expressed during growth on EPS_*Lp*_, suggesting potential activity towards the β-D-galactosyl1→3β-D-*N*-acetylglucosamine linkage present in EPS_*Lp*_.

For the GlcNAcα1 → 4Gal bond, only the GH89 family has reported activity.^47^ Here, we identified several members of the GH109 family, VK1_489, VK1_513, VK1_520 and VK1_2112. While GH109 is primarily described as α-D-*N*-acetylgalactosaminidase, considering the large quantity of GH109 and other NAD-dependent GH family members, we speculate that some may be sufficiently promiscuous to act on α-D-*N*-acetylglucosamine as well. Meanwhile, there are no known enzymes with reported activity towards the Galα1 → 3GalNAc motif and we were unable to identify a plausible candidate for the respective linkage in EPS_*Lp*_.

Lastly, our dataset also contained VK1_2210 as a uniquely expressed protein during growth on EPS_*Lp*_, a protein annotated as pyruvyltransferase and predicted to be secreted through the TAT-pathway. While this enzyme class is typically associated with the addition of pyruvate groups, its presence may reflect involvement in polysaccharide modification pathways. However, the enzymatic basis of depyruvylation remains unclear,^48,49^ and the relevance of this protein in EPS_*Lp*_ is uncertain. Nevertheless, the biochemical data (Figure 5A,B) do indeed suggest that removal of the pyruvate group is important for EPS_*Lp*_ depolymerization.

Based on these findings, we propose a starting point for a hypothetical model for degradation of EPS_*Lp*_ by *Luteolibacter* sp. VK1 (Figure 6C). Secreted GH123 family enzymes likely initiate depolymerization of EPS_*Lp*_ into oligosaccharides. The coexistence of pyruvylated and depyruvylated tetrasaccharides, coupled with the absence of fully depyruvylated higher oligosaccharides (dp5-dp8) in culture supernatants, suggests that removal of pyruvyl groups occurs terminally after depolymerization (Figure 5A), possibly involving VK1_2210. The resulting oligosaccharides are subsequently further processed by additional glycoside hydrolases, potentially including GH42 and GH109 family members, prior to uptake and metabolism. However, the precise enzymatic mechanisms and substrate specificities remain to be experimentally validated. In particular, candidate enzymes identified here will require biochemical characterization through recombinant expression and activity assays.

## Conclusion

In this study, we investigated degradation of the structurally complex glycosaminogalactan EPS_*Lp*_ and identified *Luteolibacter* sp. VK1 as an EPS_*Lp*_-utilizing strain. Biochemical assays, size-exclusion chromatography, and mass spectrometry showed that VK1 depolymerizes EPS_*Lp*_ into defined oligosaccharides, consistent with endo-acting cleavage followed by further processing.

Proteomics-driven candidate selection, supported by AlphaFold- and FoldSeek-based structural annotation, identified proteins potentially involved in EPS_*Lp*_ catabolism. Notably, a GH123 protein and a distant homologue, both carrying CBM32 domains, were detected only during growth on EPS_*Lp*_, implicating them as key candidates for extracellular EPS_*Lp*_ depolymerization.

Mass spectrometry further revealed differences in oligosaccharide pyruvylation between culture supernatants and in vitro enzyme assays. The presence of depyruvylated products under physiological conditions, but not in simplified in vitro assays, suggests that EPS_*Lp*_ catabolism involves an additional depyruvylation step whose enzymatic basis remains unresolved. The candidate proteins identified here provide a starting point for investigating this process.

Overall, this work shows how biochemical degradation assays, proteomics, and structure-based annotation can be integrated to uncover candidate enzymes involved in the degradation of complex bacterial polysaccharides. The proposed degradation model provides a foundation for future biochemical validation and expands the functional landscape of carbohydrate-active enzymes involved in microbial polysaccharide catabolism.

## Supplementary Material







## Data Availability

Genome assembly and annotations are available under Figshare. The mass spectrometry proteomics data has been deposited to the ProteomeXchange Consortium via the PRIDE (Proteomics IdentificationDatabase) partner repository with the data set identifier PXD080453 Figshare link: doi.org/10.6084/m9.figshare.32858291

## Notes

Inoculum sources used during the initial culture enrichment process (PDF)
CAZyme annotation of the *Luteolibacter* sp. VK1 genome (XLSX)
Proteomic analysis data of all proteins upregulated or uniquely detected when growing *Luteolibacter* sp. VK1 on TSB-EPS (XLSX)

## Abbreviations

CAZyme: Carbohydrate active enzyme
CBM: Carbohydrate-binding module
EPS: Exopolysaccharide
FWP: food waste processing plant
GH: glycoside hydrolase
LFQ: Label-free quantification
MALDI-ToF MS: Matrix-Assisted Laser Desorption/Ionization – Time-of-Flight Mass Spectrometry
MPL: mixed plant leaves
EPS_*Lp*_-WTA: EPS_*Lp*_-WTA mix
PL: polysaccharide lyase
SEC: Size-exclusion chromatography
TSB: Tryptic soy broth
WTA: Wall-teichoic acid
WWTP: wastewater treatment plant
