# Supplementary material for: Proteomics-Guided Discovery of Candidate Enzymes for Degradation of a Rare Pyruvylated Glycosaminogalactan by a Newly Isolated Luteolibacter Strain
Source: J Proteome Res. 2026 Sep 29;25(11):6256–66. doi: 10.1021/acs.jproteome.6c00586 (PMC13750104; doi:10.1021/acs.jproteome.6c00586)
Supplement: Supplementary file 1 [file pr6c00586_si_001.pdf]

Proteomics-guided discovery of candidate enzymes for degradation of a rare pyruvylated glycosaminogalactan by a newly isolated *Luteolibacter* strain

Victor Daisuke Kietzmann[1], Pascal Mrozek[1], G. Jacob Boehlich[1], Ronja M. Sandholm[1],

*Sabina L. La Rosa[1], Bjørge Westereng[1] and Gustav Vaaje-Kolstad[1]**

[1]Department of Chemistry, Biotechnology and Food Science, Norwegian University of Life Sciences, 1430 Ås, Norway

Email: gustav.vaaje-kolstad@nmbu.no

Table S1. (pdf) Description and origin of inoculum sources used during the initial culture enrichment process.

Table S2. (xlsx) CAZyme annotation of the Luteolibacter sp. VK1 genome and the total number of unique families identified.

Table S3. (xlsx) Proteomic analysis data of all proteins upregulated or uniquely detected when growing Luteolibacter sp. VK1 on TSB-EPS.

Table S1. Description and origin of inoculum sources used during the initial culture enrichment process.

**Identifier**	**Description**	**Location**
WWTP	Sample from anaerobic digester sludge of a wastewater treatment plant	FREVAR KF, Fredrikstad, Norway
FWP	Sample from a Food waste processing plant	FREVAR KF, Fredrikstad, Norway
Olives	Fermented olives, obtained from a local food market	Oslo, Norway
compost 1	private household compost sample	Ås, Norway
compost 2	private household compost sample	Ås, Norway
Kimchi	Home-made Kimchi, kindly provided by Prof. Geir Mathiesen	-
MPL	Mixture of life plant leave samples	NMBU campus, Ås, Norway
